# Molecular recognition in plant root endosymbiosis

**DOI:** 10.1111/tpj.71067

**Published:** 2026-08-05

**Authors:** Tora Fougner‐Økland, Maria Spezzati, Katarzyna Parys

**Affiliations:** ^1^ Genetics, Faculty of Biology LMU Munich Grosshaderner Str. 2‐4 Martinsried 82152 Germany

**Keywords:** root endosymbiosis, lysine motif receptor‐like kinase, ligand specificity, symbiosis signalling, signal transduction, SymRK, *Lotus japonicus*, *Medicago truncatula*

## Abstract

Root endosymbiosis is one of the most intimate types of plant–microbe interactions in which a symbiont is hosted within a living plant cell. In this symbiosis, nitrogen‐fixing bacteria and arbuscular mycorrhizal fungi support plant nutrient provisioning in exchange for carbohydrates and lipids. To establish these mutually beneficial interactions, plants have evolved complex surveillance mechanisms. These largely rely on the perception of microbe‐derived N‐acetylglucosamine derivatives by cell‐surface receptors that feature a lysine‐motif (LysM) ectodomain. Because these derivatives are not exclusive to symbiotic microbes, understanding how plants specifically recognise microsymbionts from the diverse array of soil rhizosphere microbiota has driven genetic, molecular and structural studies in recent years. In this review, we will summarise the current state of the art in molecular recognition by LysM receptors, provide an updated inventory of LysM receptors in land plants with annotated functionalities and highlight key aspects of signal transduction that lead to symbiont accommodation in living plant cells.

## Significance Statement

In both root nodule symbiosis and arbuscular mycorrhiza, symbiotic signalling relies on the perception of chitinous ligand by lysine motif receptors. These receptors relay the signal via SymRK, which mediates downstream signal transduction.

## INTRODUCTION

Root nodule symbiosis (RNS) with nitrogen‐fixing bacteria and arbuscular mycorrhiza (AM) with soil‐borne fungi are of high ecological importance and well‐characterised at the molecular level (Rogers & Oldroyd, 2014). Each time these interactions occur, a plant‐mediated programme guides bacteria or fungi to be hosted in a living plant root cell (Oldroyd, 2013). Given the significant energy expenditure by the plant, along with the threat from pathogenic microbes hijacking these pathways, plants employ a combined control that relies on chemical exchange between the host plant roots and the microsymbiont. This molecular dialogue culminates in plant recognition of microbial signals through a specialised family of surface lysine motif receptor‐like kinases (LysM‐RLKs) (Kelly et al., 2017). These receptors play a key role in informing plant responses, but as a group, their collective function is highly complex, with sometimes overlapping ligand specificities.

## Arbuscular mycorrhiza and root nodule symbiosis at a glance

AM is the most widespread type of terrestrial symbiosis in plants, present in around 80% of land plants (Newman & Reddell, 1987) and forms with fungi of the phylum *Glomeromycota* (Schüßler et al., 2001). The fossil record supports stable coexistence of plants and AM fungi for over 400 million years (Remy et al., 1994; Strullu‐Derrien et al., 2026), which together with the obligate biotrophy of AM fungi reflects the benefits of this association (Kiers et al., 2011). Nutrient provision lies at the heart of this relationship, facilitated by a hyphal network that extends into the soil and is specialised in the uptake of water, phosphorus, nitrogen and sulphur (Harrison, 2005). In exchange, the fungus receives lipids and photosynthetic carbon (Bago et al., 2003; Bravo et al., 2017; Helber et al., 2011; Jiang et al., 2017; Keymer et al., 2017; Luginbuehl et al., 2017). AM development begins with the formation of attachment structures on the root surface. This is followed by epidermal accommodation structures that expand through hyphal extension and branching as the fungus colonises and progresses through successive layers of host root cells (Gutjahr & Parniske, 2013). Nutrient exchange happens in the defining characteristic feature of AM, the arbuscule, a highly branched microscopic structure that forms within the root's cortical cells (Luginbuehl & Oldroyd, 2017).

In RNS, nitrogen‐fixing bacteria are accommodated within a newly developed root organ, the nodule. Nodules provide an oxygen‐deprived environment for the bacteria to fix nitrogen in a biochemical process in which atmospheric dinitrogen is converted to ammonia by the enzyme nitrogenase (Udvardi & Poole, 2013). Unlike AM, the ability to form RNS is restricted to four plant orders: Fabales, Fagales, Cucurbitales and Rosales (Soltis et al., 1995). Essentially, there are two forms of RNS. Gram‐negative rhizobia bacteria associate with the *Fabaceae* family of legumes, and *Parasponia* (Rosales) (Sprent, 2007). In contrast, actinorhizal symbiosis occurs between Gram‐positive *Frankia* and actinorhizal species (Pawlowski & Demchenko, 2012). Far more species form legume‐rhizobia symbiosis than symbioses with *Frankia*, which have largely been lost (Van Velzen et al., 2019). Around 25% of nodulating species use intercellular rhizobial infection to take up bacteria, but in most legumes, this is done through root hair infection threads (IT) (Perret et al., 2000; Quilbé et al., 2022). The IT is a tubular structure lined with plant cell wall material that encloses actively dividing bacteria as they advance toward the dividing cells of the root that will give rise to a nodule (Ardourel et al., 1994). Progression of the IT is synchronous with nodule organogenesis, ultimately leading to bacterial release and differentiation within the nodule central tissue for nitrogen fixation (Oldroyd & Downie, 2008).

The agricultural importance of legumes, together with their capacity to form AM and RNS, has motivated extensive research on the model species *Lotus japonicus*, *Medicago truncatula* and *Glycine max*. This has led to fascinating discoveries of chemical communication and early molecular events that lay the foundation of symbiotic plant‐microbe interactions.

## Bi‐directional chemical communication in root endosymbiosis

Plant survival hinges on the ability to discern beneficial from harmful microorganisms. In root endosymbiosis, this is facilitated by bi‐directional chemical communication and nutrient status. The final step in this exchange features specialised, microbe‐derived carbohydrate molecules that are sensed by the roots of the plant host.

**Figure 1:**
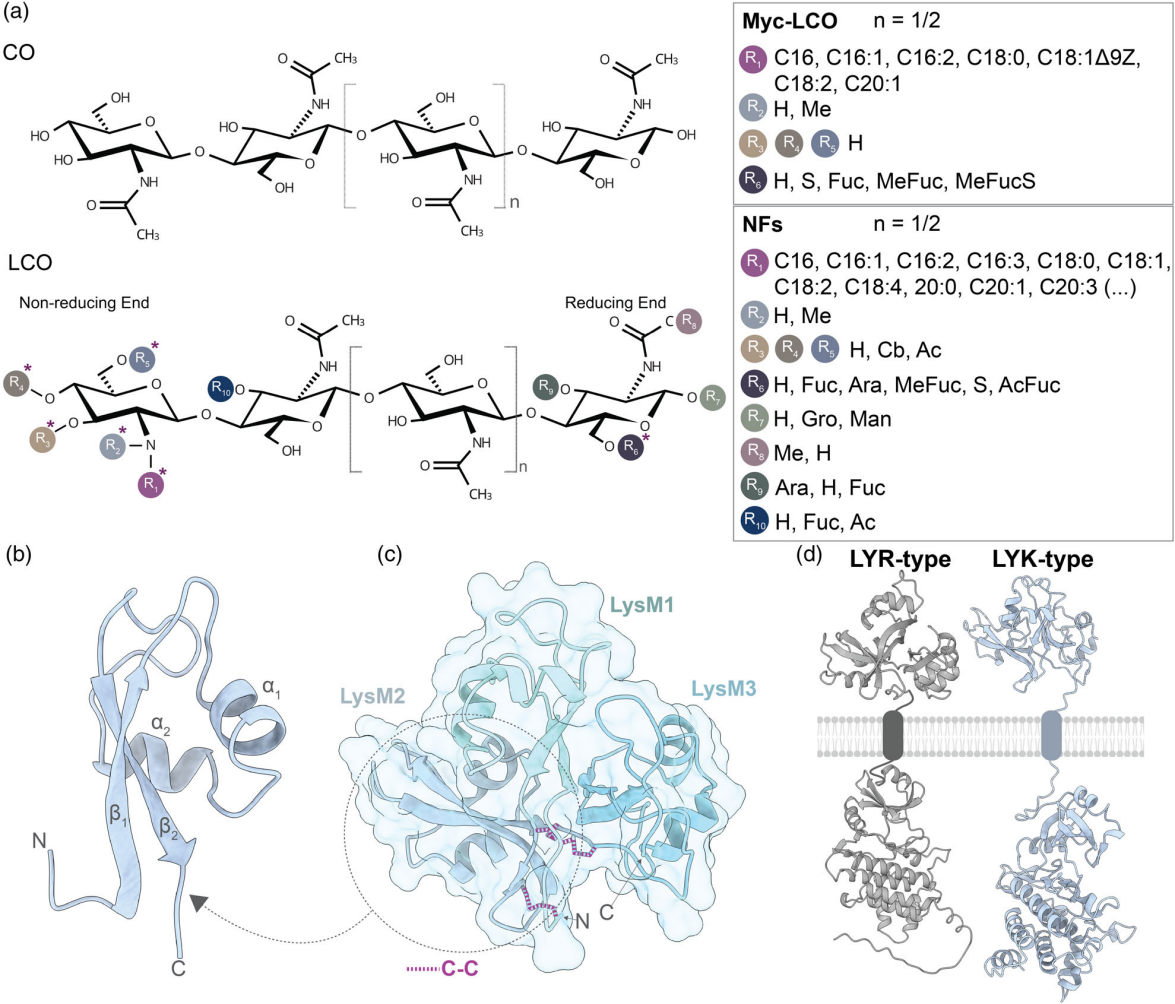
Chemically diverse chitinous ligands are detected by the lysine motif (LysM) receptors. (a) General structure of chitin oligomers (COs) and the backbone of the lipo‐chitooligosacharides (LCOs). N‐acetylglucosamine residues polymerise linearly via the β1–4 linkage. The substitutions observed at each R group are listed for Myc‐LCOs (from AM fungi) and marked with a purple asterisk or for NFs, respectively. In LCOs, an acyl moiety of varying length is attached at the R1 position of the non‐reducing end. For details on species‐specific modifications, please refer to: D'Haeze & Holsters (2002); Maillet et al. (2011); Rush et al. (2020). Ac, acetyl; Ara, arabinose; Cb, carbamoyl; Fuc, fucose; Gro, glycerol; Man, mannose; Me, methyl; S, sulphate. (b) Canonical βααβ fold structure of the LysM subunits. (c) Three LysM subunits assemble into a cloverleaf‐shaped receptor ectodomain, stabilised by three internal cysteine bridges, which are highlighted in violet. (d) Two types of LysM receptor‐like kinases are involved in COs and LCOs perception, the LYR and LYK‐type. Shown are Alpha‐Fold3 (Abramson et al., 2024) predicted structures of LYR (NFR5) and LYK (NFR1) receptors. The main difference between both types resides in the intracellular kinase domain, with alterations or deletions in conserved kinase motifs frequently found in LYR receptors. The structure of the transmembrane and juxtamembrane regions is not predicted.

Phosphate starvation stimulates biosynthesis and secretion of strigolactones (SLs) into the rhizosphere to induce AM fungi spore germination and hyphal branching, promoting the partnership (Akiyama et al., 2005; Besserer et al., 2006). A similar role of SLs in promoting AM has been reported in the bryophyte *Marchantia paleacea* (Kodama et al., 2022), making it a deeply conserved trait in land plants. In response, AM fungi release signalling compounds traditionally referred to as Myc factors (MFs), which are derivatives of N‐acetylglucosamine residues (GlcNAc) linked by a ß‐1,4 bond. There are at least two main types of MFs: fungal lipochitooligosacharides (Myc‐LCOs) (Maillet et al., 2011) and short‐chain chitooligosacharides (COs), composed of four (CO4) to five (CO5) GlcNAc residues (Genre et al., 2013; Tan et al., 2025) (Figure 1a). Chemically, LCOs resemble short‐chain COs but have a variable length lipid moiety at the terminal non‐reducing sugar and can carry other substitutions in reducing positions (Figure 1a). Myc‐LCOs were long postulated to be specific to AM fungi; however, a breakthrough discovery challenged this idea, as LCOs are secreted by diverse fungi, including pathogenic ones, and thus far, no unique features of AM‐associated Myc‐LCOs could be identified (Rush et al., 2020). Hence, other chemical cues, secretion dynamics in response to SL, or particular ratios of short‐ and long‐chain COs may be critical for specific recognition of AM and induction of symbiotic responses (Giovannetti et al., 2024). The latter is further supported by the finding that germinated spore exudates (GSEs) of AM fungi are enriched in short‐chain COs in response to GR24 treatment or low phosphate (Genre et al., 2013; Tan et al., 2025).

Various types of LCOs, known as Nod factors (NFs), are anabolically produced by rhizobia bacteria (Perret et al., 2000). NFs are the main signalling molecules in RNS, although NF‐independent symbiosis also occurs between semi‐aquatic *Aeschynomene* species and photosynthetic bradyrhizobia that lack canonical genes for NF synthesis (Giraud et al., 2007). Unlike in the plant‐AM fungi interaction, the molecular components of NF production and secretion are well‐defined and require products of *nod* genes (Downie, 1991; Schlaman et al., 1998). (Iso)flavonoids released into the rhizosphere are perceived by the NodD sensor protein (Ruan et al., 2026), triggering transcriptional activation of the *nodABC* gene cluster for the synthesis of the LCO backbone (Fisher & Long, 1992; Peck et al., 2006). The LCO backbone comprises four to five GlcNAc residues and features a characteristic acyl moiety at the non‐reducing end, with its length and saturation varying depending on the rhizobial strain. Chemical modifications are added at the non‐reducing positions, including fucosylation, N‐methylation, carbamoylation and arabinosylation (Figure 1a) (Poinsot et al., 2016). Fully decorated NFs are then secreted to target host plant roots (Spaink et al., 1995). The diversity observed in NF decorations (Figure 1a) determines rhizobia host specificity for effective nodulation (Perret et al., 2000). For example, NFs from the *M. truncatula* symbiont *Sinorhizobium melliloti* strain 2011 are both 6‐O‐sulphated and 6‐*O‐*acetylated (Roche et al., 1991). NFs without these groups have a lower ability to bind to the associated receptor in *M. truncatula* (Gysel et al., 2021), and mutant rhizobia producing these NFs display severely reduced nodulation in *Medicago sativa*, highlighting the role of NFs in determining host compatibility (Truchet et al., 1991).

## Lysine motif receptor‐like kinase family characteristics

Rhizobial NFs and fungal MFs trigger onset of plant responses including cytosolic calcium elevation, repeated calcium oscillations in the nucleus and transcriptional reprogramming. Initial recognition of signals that activate these responses is mediated by the extracellular domain of membrane proteins from the family of LysM‐RKs. The superficial similarity of COs and LCOs has long raised the question of how recognition specificity can be achieved through different members of the same receptor family. Understanding this has important implications for optimising symbiotic performance, driving research on the perception of NFs and MFs at molecular and atomic levels of resolution. Consequently, LysM receptors are among the best‐characterised receptor families in plants at the functional and structural level (Table 1). Despite this, it is a complex family to characterise, given inconsistent ortholog naming across plant species and overlapping functions (Table 1; Table S1). Here, we provide an updated systematic characterisation of this receptor family.

**Table 1:** An inventory of lysine motif receptor‐like kinases and their functional annotations

Clade name	*Lj*	*Mt*	*Mpal*	Function	Other members	References
LYK‐I	NFR1	LYK3	LYK1/LYKa Required for AM colonisation and chitin‐triggered immunity signalling.	High‐affinity NFs binding, indispensable for RNS signalling.		Bozsoki et al. (2020); Broghammer et al. (2012)
LYK‐I	LYS1/NFRe	LYK6		NFs binding; *NFRe* is expressed in the root epidermis; promotes nodule organogenesis.		Murakami et al. (2018)
LYK‐I	LYS2	LYK1		*Orphan* Can not complement *nfr1* mutant, part of a gene cluster in *Mt*.		Limpens et al. (2003); Nakagawa et al. (2011)
LYK‐I	LYS6/CERK6	CERK1 (LYK9)		Key co‐receptor in AM and chitin/PGN‐triggered immunity. AM significantly impaired in *Mt* mutant and lost in *Mpal* mutant.	*Os*CERK1	Gibelin‐Viala et al. (2019); He et al. (2019); Tan et al. (2025); Teyssier et al. (2025); Yotsui et al. (2023)
LYK‐I	LYS7	LYK8		Is required for AM colonisation and CO‐triggered symbiotic responses, but not for chitin immunity. In *Mt* interacts with CERK1 and SymRK.		Fukuda et al. (2024); Zhang et al. (2024)
LYK‐II	LYS3/EPR3	LYK10	LYKb	Recognises rhizobial EPS, is required for infection thread progression in RNS. While *EPR3* mutant partially repressed the phenotype of rhizobia EPS mutants, the opposite has been observed in *Mt*.		Kawaharada et al. (2015, 2017); Maillet et al. (2020); Wong et al. (2020)
LYK‐II	LYS8/EPR3a			*Lotus*‐specific duplication. Recognises rhizobial EPS, and beta‐glucans, is required for infection thread progression in RNS. *EPR3a* expression was observed in AM‐colonised roots, but no phenotype has been observed in AM.		Kelly et al. (2023)
LYK‐III	LYS4	LYK11	LYK2/LYKc	*Orphan*		
LYK‐III	LYS5			*Orphan*		
LYR‐IA	NFR5	NFP	LYR Required for AM colonisation and chitin‐triggered immunity signalling.	Indispensable for RNS signalling and high selectivity NF binder in legumes. Evolved upon duplication in the last common ancestor of nodulating clade. CO4 binder in non‐legumes.	*Os*MYR1/LYK2, *Sl*LYK10 In *Os* and *Sl*, promotes AM colonisation.	Arrighi et al. (2006); Buendia et al. (2016); Girardin et al. (2019); Hansen et al. (2024); He et al. (2019); Madsen et al. (2003); Radutoiu et al. (2003); Rutten et al. (2020); Tan et al. (2025); Teyssier et al. (2025); Zhang et al. (2021)
LYR‐IA	LYS11	LYR1		High affinity binding to Myc‐LCOs, AM‐associated expression but no symbiotic phenotype reported in the mutant.		Cullimore et al. (2023); Rasmussen et al. (2016)
LYR‐IB	LYS17	LYR8		Clade members display high‐affinity binding of short COs (but also binds long COs and LCOs). Mutants have reduced AM colonisation. Rice cultivar differences, natural 1 bp deletion in japonica cultivars.	*Os*LYK11, *Sl*LYK9, *Ph*LYK9, *Bd*LYR2	Ding et al. (2025)
LYR‐IIA	LYS16	LYR10		*Orphan*		
LYR‐IIB	LYS15	LYR9		*Orphan*		
LYR‐IIIA	LYS12	LYR3		High affinity binding of LCOs, but mutants do not display symbiotic phenotype. Putative modulatory role in symbiotic and immunity signalling. In *Lj*, required for resistance against *Phytophthora palmivora*. Arabidopsis ortholog and *Lupin* lost the ability to bind LCOs, strong link with AM.	*Nb*LYK4	Fliegmann et al. (2013); Fuechtbauer et al. (2018); Wang et al. (2023)
LYR‐IIIB	LYS18	LYR2		*Orphan*		
LYR‐IIIC	LYS13/CHIP13	LYR4		High affinity binding of long COs, forms complexes with CERK1/6. Required for chitin‐induced defence responses.		Feng et al. (2019); Gysel et al. (2025, preprint); Simonsen et al. (2025)
LYR‐IIIC	LYS14/CHIP14					
LYR‐IV	LYS20/LYS21	LYR5/LYR6	NA	*Orphan* Kinase is more similar to Wall‐associated kinase (WAKs) than LYRs		

*Bd*, *Brachypodium distachyon*; *Lj*, *Lotus japonicus*; *Mpal*, *Marchantia paleacea*; *Mt*, *Medicago truncatula*; NA, not available; *Nb*, *Nicotiana benthamiana*; *Os*, *Oryza sativa*; *Ph*, *Petunia hybrida*; *Sl*, *Solanum lycopersicum*.

Plant LysM domains consist of three LysM units (M1–M3) arranged in tandem in a clover‐like structure. They are structurally divergent from each other and are usually composed of a βααβ motif (Figure 1b) (Zhang et al., 2007). Three cysteine bridges stabilise the connections between the subunits (Figure 1c) (Liu et al., 2012; Zhang et al., 2007). LysM domains are found in three configurations: (i) as apoplastic protein (LysMe) or membrane‐bound via a glycosylphosphatidylinositol anchor (LYMs), (ii) featuring a transmembrane domain (LysM Receptor‐Like Proteins, LysM‐RLPs), and (iii) with a kinase domain (LysM‐RLKs) (Buendia et al., 2018). LysM‐RLKs are divided into two main functional and phylogenetic subclasses: LYKs and LYRs (Figure 1d). Since both subclasses are found across land plants, it is hypothesised that they emerged by duplication in the Most Recent Common Ancestor (MRCA) of land plants (Figure S1) (Nishiyama et al., 2018; Tan et al., 2025; Teyssier et al., 2025).

## The LYK clade

**Figure 2:**
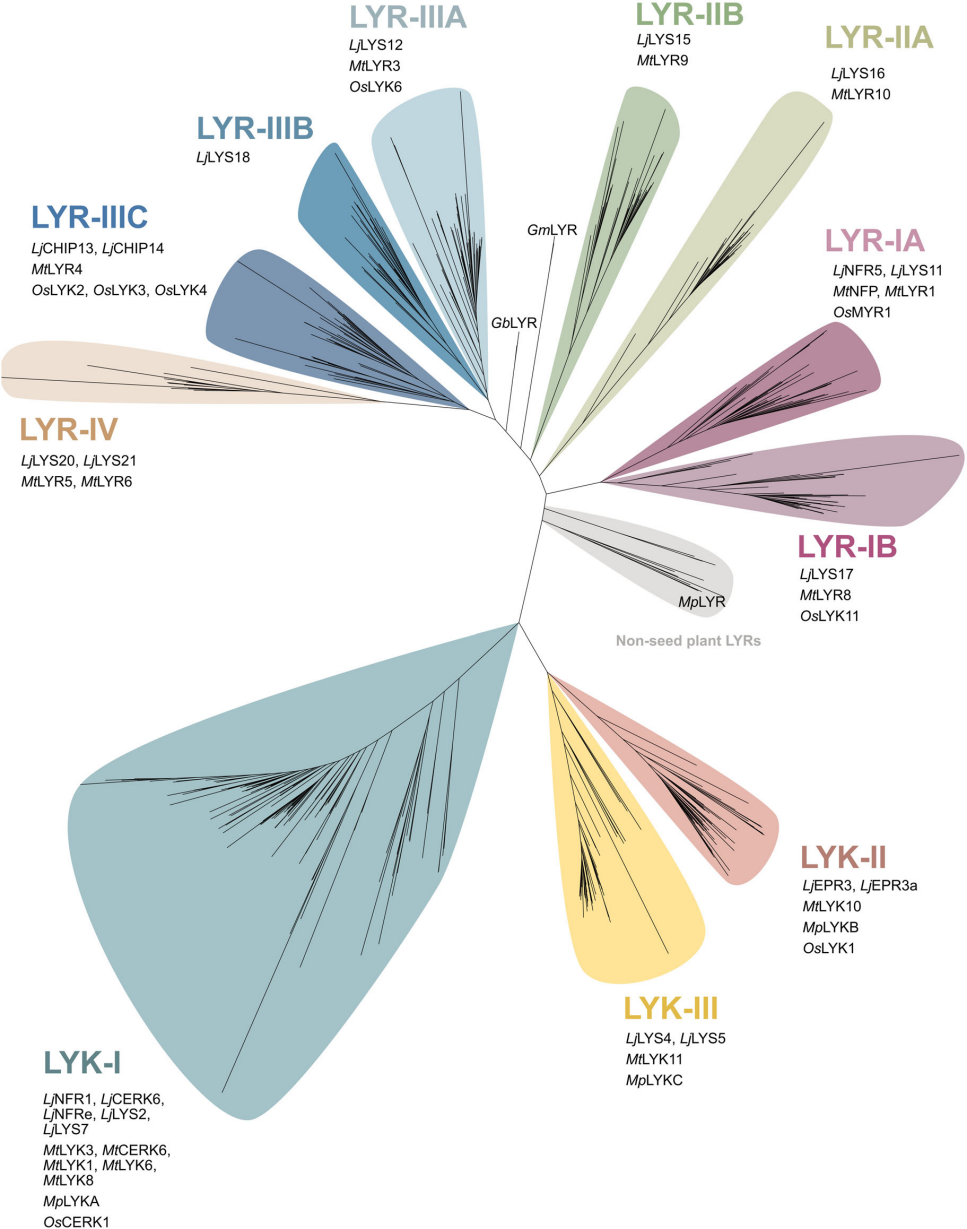
Phylogenetic tree of lysine motif (LysM) receptor‐like kinases in land plants. Eleven clearly defined LYK and LYR clades could be annotated. In addition, a group of LYRs from non‐seed plants can be seen, as well as gymnosperm LYRs that do not group within the main angiosperm LYR clades (*Gnetum montanum Gm*LYR and *Ginkgo biloba Gb*LYR). The tree was built using IQtree (Minh et al., 2020) on sequences from 47 species based on orthogroups containing *Lotus japonicus* LysM‐RLKs using OrthoFinder (Emms & Kelly, 2019) and visualised in iTOL (Letunic & Bork, 2024). Truncated genes from the identified orthogroups were retained if they included a LysM domain. Clades are highlighted with different colours and named based on (Buendia et al., 2018). Orthologs from *Lotus japonicus* (*Lj*), *Medicago truncatula* (*Mt*), *Oryza sativa* (*Os*) and *Marchantia paleacea* (*Mp*) are annotated below their specific clades.

LYKs have active kinase domains, and form three main clades in land plants (LYK I–III). This split is deeply conserved, since the three *LYK* genes in *M. paleacea* are split between the same three LYK clades found in vascular plants (Figure 2) (Tan et al., 2025; Teyssier et al., 2025). Unlike *M. paleacea*, the LYK‐I clade is expanded in angiosperms, in particular in legumes (Rutten et al., 2020). In *M. truncatula*, the LYK‐I clade includes a cluster of seven full‐length LysM receptors in the accession A17 (Limpens et al., 2003; Luu et al., 2023). Comparisons between A17 and another accession, R108, have revealed the rapidly evolving nature of this cluster in *M. truncatula*, where new genes have likely been generated through recombination of neighbouring genes. R108 carries one such additional homologue, LYK2bis, whose ectodomain is likely a chimera of LYK2 and LYK3. LYK2, LYK3 and LYK2bis appear to be functionally redundant in mediating nodulation, with LYK2bis likely playing a major role due to its higher expression levels in response to rhizobia (Li, Zhao, et al., 2024). Mutant analysis further indicates that R108 LYK2bis may act with LYK3 in NF signalling, and experiments involving expression of *LYK2bis* in other accessions suggest that variation in this LYKIA cluster contributes to host strain preferences (Luu et al., 2023).

**Figure 3:**
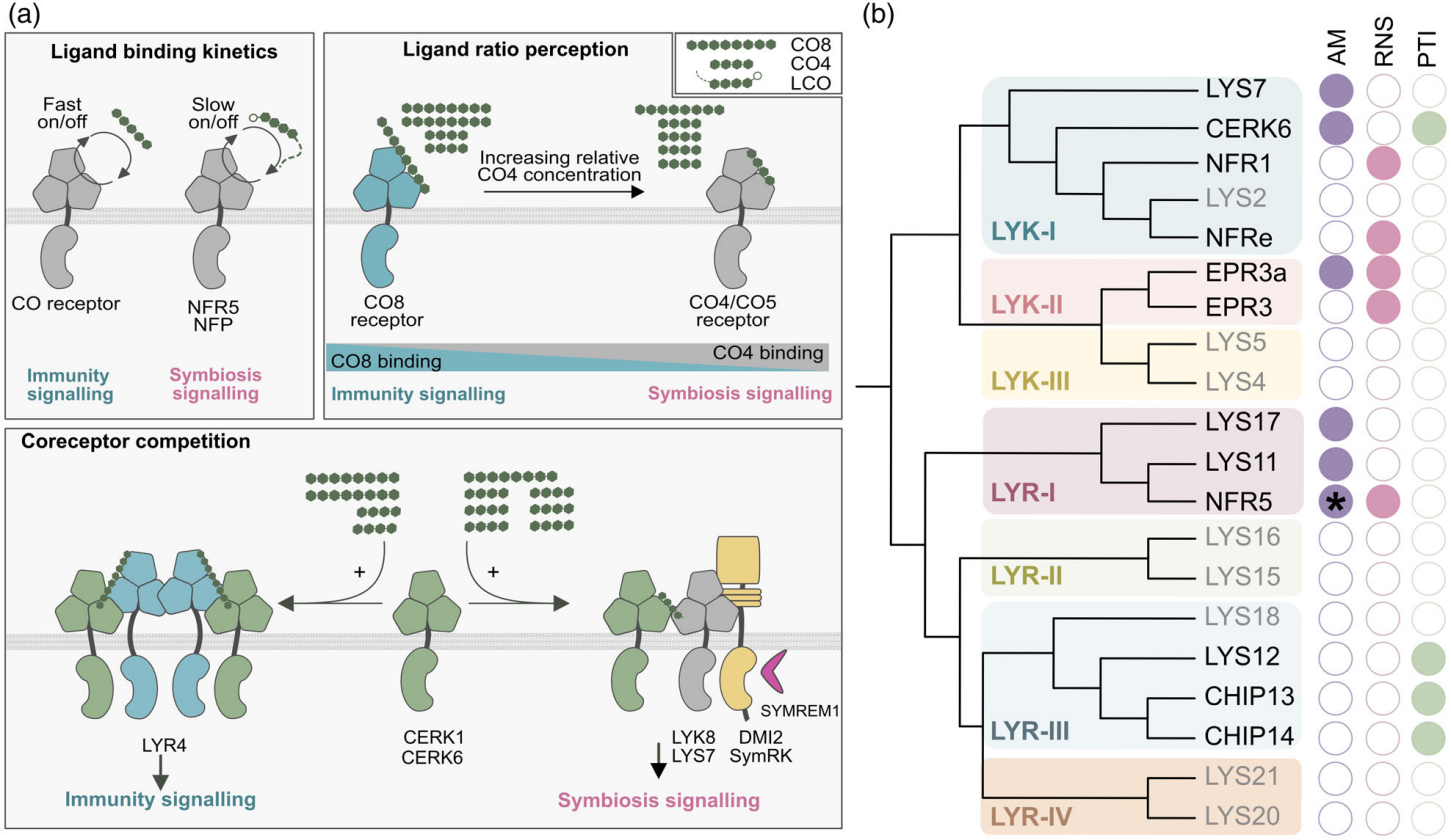
Lysine motif (LysM) receptors act at the intersection between symbiotic and immune signalling. (a) Three models of how specificity in LysM‐mediated signalling is achieved are illustrated. Upper left: Ligand binding kinetics of lipochitooligosacharides (LCOs) to NF receptors NFR5 and NFP are slow (Gysel et al., 2021), allowing time for proofreading of LCO decorations and larger complex assembly, in contrast to chitin‐binding receptors. Upper right: As the concentration of short‐chain chitin oligomers (COs) increases, proposed for AM fungi at the pre‐infection stage (Genre et al., 2013; Tan et al., 2025), more CO4/CO5 receptors are ligand‐bound, promoting symbiosis signalling. Below: the relative concentrations of long and short COs also involve competition for CERK1/CERK6 co‐receptor to form signalling complexes. Presence of long‐chain COs, perceived by LYR4 (Zhang et al., 2024) (and in rice by CEBiP and in *Lotus japonicus* by CHIP13/CHIP14; Gysel et al., 2025, preprint; Liu et al., 2016), promotes immune signalling complexes with CERK1/6. These complexes are destabilised in the presence of short‐chain COs and receptors involved in symbiotic signalling, such as LYK8/LYS7 (or MYR1 in rice; Feng et al., 2019; He et al., 2019). Recruitment of CERK1/6 and SymRK (DMI2 in *Medicago truncatula*) to these complexes promotes symbiosis signalling. (b) Tree of LysM receptor‐like kinases in *L. japonicus*, their respective LYK/LYR clades and their reported main signalling pathways. Gene family members that remain orphan in regard to function and ligand‐binding are highlighted in grey. *: *nfr5cerk6* mutants have reduced AM colonisation (Feng et al., 2019), but the phenotype of NFR5 is dependent on loss of CERK6 function. AM, arbuscular mycorrhiza; PTI: pattern‐triggered immunity, here chitin‐triggered immune responses; RNS, root nodule symbiosis.

LYK‐I members can bind both LCOs and COs of different length (Figure S3), and have functions in RNS (Nod Factor Receptor NFR1, NFRe, LYK3), AM and chitin‐triggered immunity (chitin elicitor receptor‐like kinases: *Os*CERK1, *Lj*CERK6, *Mt*CERK1) (Figure 3b; Table 1). Despite the widespread presence of LYK‐II and LYK‐III members across land plants, only a few members of LYK‐II have been functionally described: Exopolysaccharide Receptor *Lj*EPR3/EPR3a and *Mt*LYK10 (Kawaharada et al., 2017; Kelly et al., 2023; Maillet et al., 2020). Characterised receptors from this clade bind exopolysaccharides (EPS), restricting invasion of rhizobia with incompatible EPS.

## The LYR clade

In contrast, LYRs are characterised as pseudokinases and were previously classified into eight clades (Figure 2) (Buendia et al., 2018). While supported by functional data in key representatives such as *Lj*NFR5 (Madsen et al., 2011), the characterisation of LYRs as pseudokinases is based on sequence features in regions that are characteristic for kinase function. For example, in place of the conserved DFG motif in the activation loop that is required for ATP hydrolysis, LYR clade members often carry an NFG‐version of the motif. Other mutations were found in the glycine‐rich loop, the HRD motif and the activation loop (Ruman & Kawaharada, 2023). However, LYR clade members are not necessarily kinase dead. *Mt*LYR4 exhibits both autophosphorylation and transphosphorylation activity towards a generic substrate *in vitro*; however, its kinase activity is not required for its function in immunity (Simonsen et al., 2025). Thus, LYR kinase domains can have a structural, tethering role, or possibly a residual catalytic function.

The LYR‐I, II and III clades likely diverged after the emergence of seed plants, since bryophyte, lycophyte and fern LYRs group separately from these clades (Figure S1). The origin of the LYR‐IV clade is less clear, since their kinase domains share a high similarity with wall‐associated kinases (WAKs) (Figure S2; Arrighi et al. (2006)). Given the resemblance of their ectodomains to LYR clade members, this clade may represent a chimera from a recombination event between two receptor families (Figure S2). Diversification of the LYR clades in angiosperms is accompanied by sub‐functionalisation for more specific binding of short‐ or long‐chain COs and LCOs, suggesting that diversification of the main LYR clades has partially been driven by refinement of ligand recognition (Figure S4). Consistent with this, the single LYR in *M. paleacea* binds COs irrespective of their length (Tan et al., 2025). In general, LYR‐IA and LYR‐IIIA members bind LCOs with high affinity but only bind COs weakly (Cullimore et al., 2023; Fliegmann et al., 2013; Gysel et al., 2021; Wang et al., 2023), although exceptions exist: in rice, MYR1 preferentially binds short COs over NFs (He et al., 2019). The LYR‐IB clade was recently found to contain many high‐affinity short‐chain CO binders (Ding et al., 2025), while LYR‐IIIC clade members have a high affinity for long‐chain COs (Cao et al., 2014; Gysel et al., 2025, preprint). For the remaining clades (LYR‐II, LYR‐IV, and LYR‐IIIB), ligand preference is not known.

Typically, LYR and LYM proteins exhibit ligand‐binding function, whereas LYKs additionally possess a catalytically active kinase domain that is crucial for signal transduction (Arrighi et al., 2006; Buendia et al., 2018). This points to a co‐receptor function for LYKs, as suggested for other receptor families that exhibit conserved evolutionary traits (Mecchia et al., 2022; Yan et al., 2025). Fully functional signalling therefore often requires heterocomplexes of LYKs with LYMs or LYRs. In recent years, much progress has been made in characterising the molecular features that give rise to different binding capacities of LysMs and their resulting receptor complexes, improving our understanding of how signalling specificity is achieved.

## Nod factor perception in RNS

In *L. japonicus* and *M. truncatula*, NF signalling requires an active kinase from the LYK‐I clade (NFR1/LYK3) and a pseudokinase from the LYR‐IA clade (NFR5/NFP) (Amor et al., 2003; Arrighi et al., 2006; Madsen et al., 2003; Radutoiu et al., 2003). Both members bind NFs and are required for signalling, and forcing complex formation between them is sufficient to induce RNS‐specific transcription programs and spontaneous nodulation in the absence of rhizobia (Rübsam et al., 2023).

NFR1 and NFR5 perceive NFs through distinct structural features, suggesting that these receptors have undergone convergent evolution for LCO binding. Subdomain swaps between the different LysM subdomains of NFR1 and its paralog CERK1, identified LysM1 of NFR1 as highly selective for NFs (Bozsoki et al., 2020). Two regions adjacent to the putative LCO binding site are variable among legume NFR1 orthologs, especially when compared to the high conservation of the same region among CERK1 orthologs. The variability at the LCO binding site reflects the ability of legume NF receptors to distinguish between bacterial strains and species based on the decorations of their NFs. *Medicago truncatula* and *L. japonicus* form effective symbiosis with rhizobial species that produce differently decorated NFs. Consequently, *L. japonicus nfr1* cannot be complemented by native expression of *NFR1* ortholog from *M. truncatula LYK3* unless specific motifs from the LCO‐binding groove of NFR1 are swapped into the LYK3 ectodomain. Interestingly, NFR1 associates with NFs from both *S. melliloti* and its cognate symbiont *Mesorhizobium loti in vitro*, suggesting that ligand binding and receptor activation might be uncoupled (Bozsoki et al., 2020). Thus, host selectivity is at least partially imparted by structural signatures in LYK‐I NF receptors.

LYR‐IA members in legumes evolved from gene duplication in the last common ancestor of the nodulating clade (Rutten et al., 2020) and may fine‐tune NF recognition in a more discriminate manner compared to LYK‐I members. In *M. truncatula*, NFP specifically binds *S. meliloti* NFs with nanomolar affinity and lack of sulphate modifications at the reducing end alters the affinity (Gysel et al., 2021). Perception of NFs by NFP occurs via the LysM2 subdomain (Bensmihen et al., 2011; Radutoiu et al., 2007), which carries a hydrophobic patch conserved among legume orthologues (Gysel et al., 2021). It is thought that the hydrophobic surface stabilises LCO binding by anchoring the acyl chain, decreasing the dissociation rate of the ligand–receptor complex and potentially enabling a ‘proofreading’ of the side chain modifications for robust recognition. The hydrophobic patch is a signature for LCO‐binding among legume LYR‐IA members and is absent in, for example, NFR1 or LYR‐IIIA receptors, meaning that it is likely not an essential feature for high‐affinity LCO binding (Ding et al., 2025; Gysel et al., 2021). However, slower association and dissociation kinetics might be a general principle for precise recognition of NFs and activation of the receptor complex with NFR1.

Legume LYR members are likely not unique in their ability to respond to *Sinorhizobium* NFs, as mutating LYR‐IA and LYR‐IB homologues in barley led to a complete loss of calcium spiking in response to *S. meliloti* (Li et al., 2022). Although non‐leguminous species can respond to LCOs, legume NF receptors demonstrate a particularly high affinity for NFs, typically in the sub‐nanomolar range (Broghammer et al., 2012; Cullimore et al., 2023; Gysel et al., 2021). Besides strong binding, selectivity also seems to be coded in the dynamics of binding to NF receptor ectodomains. In *L. japonicus*, LYS11 is induced by AM but has no mutant phenotype in AM nor RNS (Rasmussen et al., 2016). Interestingly, LYS11 can bind *M. loti* LCOs with higher affinity than NFR5, but expression of *LYS11* under control of the *NFR5* promoter cannot complement *nfr5* mutants for nodulation (Gysel et al., 2021). This can be explained by faster association and dissociation kinetics for LYS11‐NF binding compared to NFR5. Impeding this dynamic in LYS11 enabled it to complement *nfr5* mutants. Slow binding dynamics are likely favoured in symbiosis, unlike immune responses, which require a rapid and robust reaction to quickly eliminate microbes. In symbiotic interactions, the precise recognition of NFs and the slow recruitment of larger signalling complexes reduce the risk of plants making poor decisions about accommodating microbes in these intimate relationships.

## Perception of exopolysaccharides

The epidermal uptake of rhizobia must be selective and tightly controlled by NF receptors. In a natural setting, the rhizosphere is not sterile, and pathogenic or ineffective bacteria can colonise the host alongside rhizobia (Crosbie et al., 2022). Consequently, plants possess additional mechanisms to limit the intracellular entry of these bacteria. One such mechanism involves detecting bacterial EPS, which likely serves as an extra step in determining plant–microbe compatibility during the infection stage.

EPS is produced and secreted by bacteria to facilitate surface attachment, biofilm formation or defence suppression, among other functions (Skorupska et al., 2006). This heteropolysaccharide occurs in two primary forms, depending on its degree of polymerisation: high‐ or low‐molecular‐weight EPS. Studies of rhizobial mutants affected at various stages of EPS biosynthesis have revealed the importance of EPS for IT function, in particular to IT elongation (Cheng & Walker, 1998; Kawaharada et al., 2015; Kelly et al., 2013; Pellock et al., 2000). Notably, truncated or non‐modified EPS leads to more severe phenotypes compared to mutants that lack EPS entirely. This may suggest that recognition of incompatible EPS is critical. Thus, it is proposed that rhizobial EPS exerts two roles: a static role in surface attachment or defence suppression, and signalling to the host surveillance system.

The monomeric octaose EPS is directly bound by the EPR3 and EPR3a receptors in *L. japonicus*, with μM affinity (Figure S3) (Kawaharada et al., 2015; Wong et al., 2020). Several binding sites spanning LysM1‐3 are suggested to contribute to the interaction with EPS (Wong et al., 2020). The crystal structure of the EPR3 ectodomain revealed a unique structural fold of LysM1 (βαββ) that is present in EPR3 orthologs, defining a structural hallmark of this receptor class. EPR3 receptors are present as single‐ or double copy (e.g. *L. japonicus*) in plant genomes (Kelly et al., 2023). Both EPR3 and EPR3a are required for rhizobial infection progression in the epidermis but have distinct phenotypes in AM accommodation (Kawaharada et al., 2015; Kelly et al., 2023). *EPR3* is predominantly expressed in the epidermis, and its expression is induced by NFs, indicating signalling activity different to and downstream of NF perception (Kawaharada et al., 2015, 2017). In contrast, *EPR3a* shows AM‐associated expression in the inner cortical cells and promotes AM (Kelly et al., 2023). The extracellular matrix of AM fungi contains beta‐glucans, and EPR3 and EPR3a bind to *Serendipita indica* β‐1,3/β‐1,6 glucans (Kelly et al., 2023). This suggests that EPR3 is likely a general EPS/β‐glucan surveillance receptor, supported by the extensive diversity observed in EPS produced by rhizobia and the high promiscuity of the receptor. EPR3 binds EPS derived from *M. loti*, but also *S. meliloti*, and *Rhizobium leguminosarum* that do not nodulate *L*. *japonicus* (Kelly et al., 2023). This could indicate that EPR3 monitors the EPS status of bacteria in a generalist, rather than a specific or symbiotic, context. For example, *S. meliloti* produces succinoglycan, but in *M. truncatula*, LYK10 (the ortholog of EPR3) does not appear to be involved in succinoglycan perception (Maillet et al., 2020). This highlights possible species‐specific differences and surrogate receptors involved in EPS recognition in legumes.

## Structurally similar molecules activate symbiosis and immune signalling

Symbiotic and immune signalling share molecular features but lead to opposite outcomes (Zipfel & Oldroyd, 2017). These divergent outcomes can be driven both by the specificity of ligand perception by the ectodomain and by intracellular sequence signatures that underlie specificity in signal transduction. The latter is exemplified by sequence determinants located in the juxtamembrane regions of the NFR1 and NFR5 receptors, which underpin their specific responses during RNS activation (Hansen et al., 2024; Tsitsikli et al., 2025).

Signalling specificity is less understood in AM symbiosis, including at the levels of microbial signatures and plant perception. LCOs are not exclusive to AM fungi (Rush et al., 2020). Instead, a mix of COs and LCOs is found in germinated spore exudates of AM fungi. They trigger symbiotic outputs, including nuclear calcium oscillations, suggesting a role in molecular dialogue at the pre‐infection stage of AM (Genre et al., 2013; Maillet et al., 2011; Tan et al., 2025). On the other hand, COs are also perceived as defence molecules by plants and are referred to as microbe‐associated molecular patterns (MAMPs), as they induce a series of defence responses collectively known as pattern‐triggered immunity (PTI) (Zipfel & Oldroyd, 2017). Therefore, the recognition of symbiotic fungi is complex, relying on the perception of a mix of LCOs and COs with different chain lengths to balance symbiotic and immune response activation. Since these molecules function as both symbiotic and immune signals and are recognised by members of the same receptor family, a central question in symbiosis signalling is how plants discriminate between pathogenic and symbiotic fungi.

Simplistically, short‐chain COs (e.g. CO4) and Myc‐LCOs promote symbiotic signalling by inducing nuclear calcium oscillations and symbiotic gene expression (Feng et al., 2019; Genre et al., 2013; Sun et al., 2015). In contrast, long‐chain chitin oligomers (e.g. CO8) are known to elicit PTI responses (Cao et al., 2014; Feng et al., 2019; Stacey & Shibuya, 1997). In this model, a binary perception mechanism is proposed to discern AM and pathogenic fungi (Giovannetti et al., 2024). This model, however, is far from parsimonious because short‐chain COs can stimulate immune responses, although CO8 is more potent. Conversely, long‐chain chitin oligomers and peptidoglycan, both immunogenic, can also induce nuclear calcium spiking in *M. truncatula* (Feng et al., 2019). These findings prompted studies of plant responses to a mix of chitin‐based molecules that closely replicate a natural setting. Interestingly, coordinated perception of long‐ and short‐chain COs or LCOs leads to suppression of defence outputs, such as reactive oxigen species (ROS) responses and MAPK activation, promoting symbiotic signalling (Feng et al., 2019; Tan et al., 2025; Zhang et al., 2021). In this scenario, competition could arise from differences in relative concentration of different chitin‐derived molecules, including those that suppress defence responses and support AM fungi accommodation (Giovannetti et al., 2024).

Suppression of plant defences by short‐chain COs would be an effective strategy to promote symbiosis if only symbiotic fungi, and not pathogenic fungi, could produce them in excess. While LCOs are secreted by fungi of all types, strong enrichment of short‐chain COs has been observed for symbiotic fungi when exposed to GR24, an analogue of SLs, or media in which plants were grown under low phosphate conditions (Genre et al., 2013; Tan et al., 2025). Short‐chain COs were also detected in the GSEs of pathogenic fungi, but neither the GSEs nor the external addition of CO4 to these GSEs induced calcium oscillations in *M. truncatula* roots, suggesting the presence of inhibitory molecules or competing factors (Genre et al., 2013). In line with key differences in AM fungi responses to plant signals, or mechanisms specific to these fungi that lead to increases in active short‐chain COs, Tan et al. (2025) revealed that GR24 treatment did not induce CO4–CO5 enrichment in pathogenic fungi. This suggests that pathogenic fungi may be unable to respond specifically to SLs with increased short‐chain CO production. One plausible explanation for the relative increase in short‐chain COs is the context‐specific induction of plant and/or fungal chitinase activities mediating the degradation of long‐chain COs (Feng et al., 2019; Salzer et al., 2000; Schmitz & Harrison, 2014). In this scenario, plants secrete SLs when phosphate is scarce, which then locally increases CO4/CO5 levels from AM fungi, and this local increase is decisive for uptake by the plant (Volpe et al., 2023). Transcriptional regulation enhances this feedback loop, since phosphate starvation also increases expression of LysM receptors and core symbiotic genes, promoting symbiotic signalling (Das et al., 2022). In this way, changes in relative CO4/CO5 concentrations and transcriptional responses could together shift signalling in favour of symbiotic outcomes (Volpe et al., 2023).

## Chitin oligomer perception is ancestral in land plants

CO and LCO perception and response in AM symbiosis involves an interplay between LysM receptors that perceive both signals. Hence, some LysM receptors have a dual functionality in chitin‐triggered immunity and AM symbiosis, consistent with context‐specific recruitment of LysM‐RLK interaction partners.

In angiosperms, CO‐mediated symbiosis and immune signalling share overlapping LysM receptor repertoires, and the expansion among the LYR clades makes it difficult to dissect their individual roles. *Marchantia paleacea* has drastically fewer LysM receptors than most angiosperms, simplifying efforts to dissect the functional roles of individual receptors. It maintains sensitivity to short and long‐chain COs and LCOs (Teyssier et al., 2025), indicating that chitinous perception by LysM receptors is highly conserved in land plants. Mutant analysis in *M. polymorpha*, a species that has lost AM, identified LYK1 and LYR as required for CO7‐ and peptidoglycan‐triggered responses (Yotsui et al., 2023). This is consistent with the role of members of this clade in the perception of long‐chain COs in angiosperms (Table 1). In *M*. *paleacea*, the LYK1 ortholog, referred to as LYKa or CERK1, is also required for AM colonisation. *Lyka* mutants are the only described single LysM mutants that display a total loss of colonisation in AM, suggesting that the sub‐functionalisation and redundancy observed in angiosperms is a derived trait (Teyssier et al., 2025). Together, this suggests that the intersection between symbiosis and defence signalling is ancestral, and chitinous ligand recognition was likely essential for the evolution of AM symbiosis. Since the same receptor pair controls opposite outcomes, the molecular basis of how specificity is achieved is a compelling question.

In *M. paleacea*, LYR binds to different length COs and forms a heterocomplex with CERK1 (LYK1/LYKa) (Tan et al., 2025; Teyssier et al., 2025). This aligns with reports that LYR‐type receptors are high‐affinity binders of COs, and that CERK1 likely has a co‐receptor function (Yang et al., 2022). The mechanism by which LYR can discriminate between different ligands can potentially rely on excess‐ligand competition in a symbiotic context. This is consistent with the several‐fold increase in CO4/CO5 abundance relative to long‐chain COs in germinated AM fungi spore exudates in low‐phosphate conditions or after treatment with a synthetic SL analogue GR24, and low selectivity of LYR for COs or LCOs (Genre et al., 2013; Tan et al., 2025). Accordingly, CO4 marginally activates the defence transcriptional program and inhibits ROS responses induced by CO7, while adding CO7 to the growth media reduces AM colonisation (Tan et al., 2025). This is not the case for LCOs, which might suggest that LCOs cannot outcompete long‐chain COs for receptor binding, or that a high‐affinity LCO receptor is lacking in *M. paleacea*. Since ROS and AM responses are suppressed by different length COs, receptors likely experience continuous ligand competition at the pre‐infection stage, where increases in short COs from spore exudates saturate the receptor binding sites promoting symbiotic signalling. In addition, lack of a clear preferred LYR ligand suggests that the ancestral LYR had general CO‐LCO binding activity, and the expansion of the LYR subclades may have enabled discriminate recognition of symbionts through more nuanced ligand recognition.

## CERK1 at the interface of immunity and symbiosis

The ancestral role of CERK1 (LYKa) in *M. paleacea* positions the receptor at the interface between symbiotic and defence signals. Similarly, CERK1 in rice is required for chitin‐triggered immune responses and symbiotic signalling, although unlike *M. paleacea*, AM colonisation is not entirely abolished in *cerk1* mutants (Carotenuto et al., 2017; He et al., 2019; Miyata et al., 2014; Zhang et al., 2015). CERK1 orthologs characterised in angiosperms do not have canonical ligand‐binding activity, but act as a co‐receptor for high‐affinity CO‐binders. In rice, CERK1 associates with Chitin Elicitor Binding Protein (CEBiP), which is a LysM receptor‐like protein, and MYR1 (Shimizu et al., 2010; Zhang et al., 2021). CEBiP is the major CO‐binding receptor and requires CERK1 for chitin‐induced immunity and defence against *Magnaporte oryzae* (Kaku et al., 2006; Miyata et al., 2014; Shimizu et al., 2010; Zhang et al., 2015). MYR1, an ortholog of *Lotus* NFR5, is required for AM colonisation and suppression of CO8‐induced defence signalling by CO4 (Zhang et al., 2021). Tomato and *Petunia* LYR‐IA members were also reported to play a role in AM accommodation (Buendia et al., 2016; Girardin et al., 2019). Partner choice for CERK1 in rice is broadly determined by the ligand length. In the presence of CO4 and MYR1, the CERK1‐CEBiP complex becomes destabilised, likely to promote symbiotic signalling. Conversely, CEBiP or CO8 reduces the recruitment of CERK1 to the complex with MYR1, thereby inhibiting symbiosis activation (Zhang et al., 2021). It is therefore proposed that active competition for the CERK1 co‐receptor in rice serves as a gateway for the activation of symbiotic or immune signalling (Chiu & Paszkowski, 2021). While this mechanism offers a model, open questions surround the reported binding of CEBiP to both CO4 and CO8 (Liu et al., 2016). Nevertheless, these dynamics support the hypothesis that CERK1 and its orthologs act as co‐receptors, shuttling between complexes and mediating the output toward either immunity or symbiosis. In reference to the ligand competition observed in *M. paleacea*, this illustrates how receptor complex diversity may enable more discriminate symbiont recognition in AM.

CERK1 is part of the LYK‐I clade, with characterised orthologs LYK9 in *M. truncatula* (also known as CERK1) and CERK6 in *L. japonicus* (also known as LYS6). Legume CERK1 and CERK6 also have a dual role in AM and immunity (Bozsoki et al., 2017; Fukuda et al., 2024; Gibelin‐Viala et al., 2019). A striking difference to rice CERK1 is the reported binding activity of the CERK6 ectodomain to long‐chain chitin oligomers (Bozsoki et al., 2017). As only very weak binding to CO5 was detected using an orthogonal biophysical approach in Gysel et al. (2025, preprint), this suggests that *in vitro* ligand binding studies are prone to picking up interactions outside a physiological range. Legumes have a paralog of CERK‐type receptors, namely LYK8 in *M. truncatula* and LYS7 in *L. japonicus*. The single mutant shows a reduced AM phenotype that becomes more severe in the double mutant of *cerk1/lyk8* (*lys7/cerk6* in *Lotus*), suggesting genetic redundancy between paralogs (Fukuda et al., 2024; Zhang et al., 2024). As *Medicago* LYK8 is dispensable for chitin immunity (Zhang et al., 2024), this suggests LYK8 has specialised to promote symbiotic signalling. The AM‐promoting activity of LYK8 is not attributed to ligand binding, but ligand‐supported interaction with CERK1. In other words, CERK1 forms context‐dependent signalling modules through complex formation with LYR‐ and LYK‐I type receptors.

## LYR‐type receptors modulate CERK1 signalling output in symbiosis and immunity

The reliance of plants on a single convergent receptor, such as CERK1, to make opposing decisions, may offer more adaptable responses. To mediate chitin‐triggered defence, CERK1 associates with high‐affinity long‐chain COs binding partners such as Arabidopsis LYR5, *L. japonicus* LYS13/14 (tandem duplicated, also known as CHIP13/14) and *M. truncatula* LYR4 (Cao et al., 2014; Gysel et al., 2025, preprint; Simonsen et al., 2025). *Medicago truncatula* LYR4 is required for CO‐triggered defence responses and *Botrytis* resistance, similar to CERK1. Notably, the chitin‐triggered ROS response is almost completely abolished in the LYR4/CERK1 double mutant (Simonsen et al., 2025). In *L. japonicus*, high‐affinity CO8 binding is achieved by LYS13/14 and promotes CERK6 recruitment, and high‐molecular‐weight complexes may potentiate the signalling (Gysel et al., 2025, preprint). Structural studies of this complex with CO8 revealed a distinct ‘bridge domain’, which assists in size‐selective CO8 binding along with the LysM2 and LysM3 subdomains. These findings suggest that LYR‐IIIC receptors act as scaffolds for CERK1 recruitment during chitin immunity.

In *M. palecea* CERK1 pairs with LYR, while in rice a CERK1‐MYR module is involved in AM fungi perception (He et al., 2019; Tan et al., 2025). CERK1 coupling with legume LYR receptors for AM signal recognition is less well understood. Overall, LYR‐I clade receptors have high affinities for LCOs and short COs, suggesting that they function in AM. Members of the LYR‐IB subclade strongly bind LCOs and short COs and promote AM colonisation in *Solanum lycopersicum*, *M. truncatula* and *Petunia hybrida* (Ding et al., 2025). Conversely, strong expression of the *Brachypodium distachyon* ortholog in wheat increases AM colonisation. Members of the LYR‐IA subclade, including rice MYR1, *L. japonicus* NFR5 and LYS11 bind LCOs with high affinity. LYR‐IA also exclusively contains members from species that can engage in root endosymbiosis, further supporting the hypothesis that they primarily function in symbiosis signalling. Interestingly, Feng et al. (2019) observed that mycorrhization is not altered in the *Medicago nfp* mutant, but the double *cerk1/nfp* mutant is drastically less colonised by AM fungi than the single *cerk1* mutant. Given that NFP is also required for CO8‐induced defence suppression by symbiotic LCOs, and NFP contributes to defence against the oomycyte *Aphanomyces enteiches* (Rey et al., 2013), the reduced AM colonisation in the double mutant is likely due to imbalanced signal recognition and inadequate defence suppression, both necessary for microbe accommodation. This mirrors MYR1‐related observations in rice and suggests that ligand‐induced receptor competition is also present in legumes. Accordingly, LYR‐type receptors with different ligand affinities compete to associate with CERK1, a potent receptor‐like kinase for signal transduction, placing CERK1 as a signalling hub in the immunity versus symbiosis conundrum. This makes CERK1 and its orthologs a key target for future engineering.

## AM‐associated receptor modules

If LYR‐type receptors predominantly function as ligand‐selective competitors and thereby contribute to shifting the balance between immunity and symbiosis, understanding which other receptors besides LYR‐I are needed for AM becomes critical. Patterns of gene retention associated with the host's AM‐symbiotic status, along with the genes lost in species that no longer retain AM, may offer insights into this question (Bravo et al., 2016; Delaux et al., 2014; Radhakrishnan et al., 2020). For example, the LYK‐II clade, which includes legume EPS receptors, contains members found only in species capable of forming symbiotic relationships; no full‐length members have been identified from species that do not engage in AM or RNS (Figure S1) (Ruman & Kawaharada, 2023; Teyssier et al., 2025). However, AM and RNS species can evidently also lose genes from this clade, since *Parasponia andersonii*, a nodulating species, also has no functional gene in this clade (Dupin et al., 2022). The LYR‐IIB clade similarly lacks members from non‐AM species, including *Lupinus albus*, a legume that can engage in RNS but not AM (Oba et al., 2001). Members of this clade remain orphan in terms of function and ligand bound, illustrating understudied aspects of the LysM receptor family.

Strong association with AM symbiosis has been postulated for the LYR‐IB and LYR‐IIIA clade receptors. It was thought that the LYR‐IB clade only contained members from mycotrophic plants, aligning with the clade's propensity for high‐affinity short COs binding and reduced AM colonisation in the corresponding mutants in *M. truncatula*, *P. hybrida*, *B. distachyon*, tomato and rice (Ding et al., 2025). However, this hypothesis requires clarification as LYR‐IB members are found in the non‐AM species *Beta vulgaris*, *Gnetum montanum* and *L. albus* (Figure 2; Figure S1). LYR‐IIIA members seem to have different roles in modulating symbiotic and immunity signalling (Wang et al., 2023). The capacity to perceive LCOs by LYR‐IIIA receptors seems to be predominantly associated with AM species (Fliegmann et al., 2013; Malkov et al., 2016; Wang et al., 2023). Both legume and solanaceous members were shown to bind LCOs and suppress defence responses, facilitating AM (Wang et al., 2023). This capacity is lost in species that lost AM: *Arabidopsis thaliana* LYK4 and its ortholog in *Lupin* species cannot bind LCOs (Malkov et al., 2016; Wang et al., 2023). These observations potentially suggest that LCO binding in this clade is a specific feature associated with AM symbiosis signalling. This specificity is especially interesting given the observation that *NbLYK4‐*silenced tobacco plants displayed higher infection and colonisation rates and LYS12 in *L. japonicus* is required for resistance against *Phytophthora palmivora* (Fuechtbauer et al., 2018; Wang et al., 2023). The observed associations between LYR‐IIIA and LYK‐I members (Fliegmann et al., 2016; Xue et al., 2019) suggest that the ligand perceived and co‐receptor context determine which outputs are observed in plant‐fungi interactions.

## LysM‐mediated signal transduction in root endosymbiosis requires SymRK

Extracellular detection of symbiotic signals by LysM receptors and activation of receptor complexes initiates intracellular signal transduction, marked by high‐frequency calcium oscillations in the nucleus. Activation and decoding of nuclear calcium spiking depend on a set of conserved common symbiosis genes (CSG) (Kistner et al., 2005; Kistner & Parniske, 2002). The products of these genes are directly or indirectly required for cellular reprogramming and microbial uptake, and their mutants display aborted infection attempts or a complete absence of epidermal infection structures (Parniske, 2008). Key products of CSGs required for the activation of nuclear calcium spiking include membrane‐bound symbiosis receptor‐like kinase (SymRK) (Endre et al., 2002; Stracke et al., 2002), nuclear cation channels and pore components (Ané et al., 2004; Charpentier et al., 2016; Imaizumi‐Anraku et al., 2005; Kanamori et al., 2006; Saito et al., 2007). Subsequently, a probable decoder, calcium calmodulin‐dependent protein kinase (CCaMK) (Lévy et al., 2004; Mitra et al., 2004), phosphorylates and forms a complex with CYCLOPS, a transcription factor required to activate the transcriptional network for symbiotic infection (Horváth et al., 2011; Messinese et al., 2007; Singh et al., 2014; Yano et al., 2008). SymRK functions as the most upstream‐shared genetic component of the symbiotic signalling (Hayashi et al., 2010; Ried et al., 2014; Rübsam et al., 2023). In addition, SymRK promotes symbiosis by suppressing flagellin‐triggered immune responses (Feng et al., 2021). This positions SymRK as a gatekeeper for the activation of symbiotic signalling in AM and RNS.

SymRK features a malectin‐like domain, except in some commelind species, and two to three leucine‐rich repeats linked via a transmembrane helix to an intracellular kinase domain (Markmann et al., 2008; Miyata et al., 2023). Genetic and interaction studies using ubiquitous promoter‐driven expression confirm that SymRK associates with LysM receptors and acts downstream of them, reinforcing its co‐receptor role in symbiotic signalling (Antolín‐Llovera et al., 2014; Miyata et al., 2023; Ried et al., 2014; Zhang et al., 2024). SymRK interacts with NFR1 and NFR5, and the interaction is likely not affected by exogenously added NFs (Ried et al., 2014). SymRK malectin‐like domain (MLD) release further enhances the SymRK‐NFR5 interaction (Antolín‐Llovera et al., 2014). Hence, the MLD serves as a steric inhibitor, likely influencing the assembly and stability of the SymRK‐NFR5 complex, and possibly other complexes with LysM receptors involved in AM (Antolín‐Llovera et al., 2014; Kosuta et al., 2011). Preventing MLD release through mutations hinders SymRK's role in IT formation and progression to cortical cells of the nodule (Antolín‐Llovera et al., 2014; Chakrabarti et al., 2026). Since co‐expression of MLD alone mitigates these phenotypes, SymRK ectodomain cleavage serves functions beyond merely relieving the steric hindrance.

The co‐receptor role of SymRK with LysM receptors in AM is more ambiguous. In rice, SymRK interacts with *Os*CERK1 (Miyata et al., 2023), while in *Medicago*, binary interactions between LYK8‐CERK1 and LYK8‐SymRK are enhanced by CO4/8 (Zhang et al., 2024). This enhancement suggests that triple or higher order complexes may foster AM symbiosis signalling. Thus, receptor complexes containing SymRK could determine symbiotic specificity. The different effects of NFs and COs on these interactions remain unclear. Notably, all these interaction studies have used protein overexpression systems, often in *Nicotiana benthamiana* leaves, and not *in vivo* interaction studies in plant roots under native settings.

Perception and response to symbiotic signals are likely mediated through hetero‐multimer complexes assembled within specific nanodomains in the membrane, rather than through a simple binary interaction (Lefebvre et al., 2010; Liang et al., 2018; Tóth et al., 2012; Zhou et al., 2024). Membrane scaffold proteins such as remorins and flotilins support this organisation, forming *M. loti*‐dependent signalling units. Higher order complexes may arise from self‐oligomerisation or heterotypic complexes. For instance, NFR5 can oligomerise in a ligand‐dependent manner via a hydrophobic juxtamembrane motif in its intracellular domain. While necessary for receptor function, self‐oligomerisation was insufficient to initiate nodule organogenesis (Hansen et al., 2024). Additional regulatory elements include RINRK1 (rhizobial infection receptor‐like kinase 1), an LRR receptor that associates with the ectodomains of NFRs (Li et al., 2019; Zhou et al., 2024), or ubiquitin ligases that modulate NFR1/5 and rice CERK1 protein homoeostasis in a signal‐ and phosphorylation‐dependent manner, thereby modulating symbiosis and immune signalling competence (Li, Ou, et al., 2024; Wang et al., 2024).

## Cytoplasmic signalling downstream of SymRK

Signal transduction in plant pattern‐triggered immunity and developmental signalling depends on phosphorylation‐driven activation of, among others, cytoplasmic kinases and mitogen‐activated protein kinases (MAPKs), triggering rapid responses that amplify the signal to the nucleus (Zipfel & Oldroyd, 2017). In symbiotic signalling, the pathway is less well‐defined, prompting questions about whether canonical signalling components are involved or whether different types of connectors bridge membrane signalling to nuclear calcium spiking.

Individual partners that interact with NF receptors and SymRK have been identified, including the transcription factor SIP1 (SymRK interacting protein 1) (Zhu et al., 2008) and the MAPKK SIP2 (Chen et al., 2012). These proteins may link plasma membrane signalling to nuclear transcriptional reprogramming, but their mutants show only a minor decrease in symbiotic accommodation. Additionally, several cytoplasmic kinases are involved. NICK4 (NFR5‐iinteracting cytoplasmic kinase 4) interacts with and phosphorylates NFR5, and relocates to the nucleus following NF treatment (Wong et al., 2019). However, its mutants exhibit only a slight decrease in nodule number, possibly due to genetic redundancy. In *M. truncatula*, LICK1/2 (LYK3‐interacting cytoplasmic kinases 1 and 2) associate with both DMI2 (*M. truncatula* SymRK) and LYK3, functioning as reciprocal phosphorylation substrates for LYK3 (Wang et al., 2025). This promotes symbiotic signalling and contributes to immune suppression, exemplifying a fine‐tuned mechanism in which the same kinase network can simultaneously activate symbiosis and suppress defence. In AM, cyclin‐dependent kinase‐like (CKL1/2) proteins, which are phosphorylated by SymRK and some LysM receptors, regulate the arbuscule lipid provisioning transcriptional program, which is essential for arbuscule formation (Ivanov & Harrison, 2024). Notably, CKLs facilitate transcriptional activation in cortical cells that bypasses CCaMK. This suggests that transcriptional programs in the epidermis and cortical cells might be activated by different signalling components, depending on tissue context and the developmental complexity of symbiosis.

Auto‐active CCaMK restores nodule organogenesis in *symrk* mutants, suggesting that SymRK's primary function is to activate nuclear calcium spiking (Hayashi et al., 2010; Miwa et al., 2006), even though the underlying mechanism is unknown. SymRK's kinase activity is indispensable for its function (Yoshida & Parniske, 2005), thereby linking its role as a co‐receptor to the activation of the downstream signal transduction pathway. Several kinase substrates have been identified (Choudhury & Pandey, 2024; Fu et al., 2023; Ivanov & Harrison, 2024; Jayaraman et al., 2017; Lefebvre et al., 2010; Liu et al., 2018; Madsen et al., 2011; Roy Choudhury & Pandey, 2022; Tóth et al., 2012; Vernié et al., 2016). One such substrate, early phosphorylated protein 1 (EPP1) (Ferrer‐Orgaz et al., 2024; Valdés‐López et al., 2019), is docked onto SymRK via discrete structural rearrangements (based on alpha fold 3 predictions) in the kinase domain last α‐helix triggered by auto‐phosphorylation (Abel et al., 2024; Nørgaard et al., 2025, preprint). The critical function of these auto‐phosphorylation sites has been established in RNS (Abel et al., 2024). Trans‐phosphorylation of EPP1 is a conserved mechanism in plants and is present in *M. paleacea*, and is required for microbial accommodation in both symbioses (Nørgaard et al., 2025, preprint; Rich et al., 2025, preprint). While EPP1 is a direct downstream of SymRK molecule required for calcium oscillations (Valdés‐López et al., 2019), its precise molecular role and direct link to nuclear calcium oscillation remain unclear. Besides EPP1, 3‐hydroxy‐3‐methylglutaryl CoA reductase 1 (HMGR1) has long been considered a key suspect in the progression of symbiotic signalling to the nucleus (Kevei et al., 2007). SymRK ortholog in *Medicago* (NORK) interacts with HMGR1, a key enzyme in the mevalonate pathway, suggesting a connection between nodulation signalling and the biosynthesis of isoprenoid‐derived metabolites. Given that mevalonate can induce nuclear calcium oscillations (Venkateshwaran et al., 2015), HMGR1 may provide the direct missing membrane‐to‐nuclear signalling link. Key questions remain unresolved: (i) where in the cell this interaction occurs, (ii) how mevalonate (or other metabolites) biosynthesis is regulated, and (iii) which protein is targeted by mevalonate.

A range of intracellular SymRK interactors have been characterised as either promoting or negatively affecting symbiotic signalling, or involved in immune responses (Den Herder et al., 2012; Feng et al., 2021; Liu et al., 2018; Vernié et al., 2016; Yamazaki et al., 2022; Yuan et al., 2012). This demonstrates the multifunctionality of SymRK. Notably, because most interactions have been identified using yeast‐two‐hybrid assays, which may overlook dynamic, phosphorylation‐dependent interactors, our understanding of signal transduction in root endosymbiosis remains limited. Of particular interest are phosphorylation‐guided interactions: CO4‐specific phosphorylation on SymRK, absent with CO8 (Tan et al., 2025), suggests that the intercellular domain of SymRK serves as a specific docking platform for activating signalling events (Nørgaard et al., 2025, preprint).

## CONCLUDING REMARKS

The LysM‐RK family has evolved expanded recognition specificities that promote root endosymbiosis with nitrogen‐fixing bacteria and AM fungi, while simultaneously restricting pathogen growth. The functional duality likely represents an ancestral trait that has expanded in angiosperms, particularly in legumes, to enable a finer balance between activation of symbiotic and immunity signalling.

LysM‐RKs perceive at least four classes of molecules: rhizobial NFs and EPS, fungal LCOs and COs and are modulated by complex mechanisms. The similarity in how different GlcNAc‐derived signals are perceived is likely balanced by honed differences in ligand‐binding dynamics and receptor complex assembly (Figure 3a). Recognition specificity can arise from chemical modifications, varying lengths and relative abundance of chitin oligomers, as well as physical properties such as solubility (Giovannetti et al., 2024). Binding dynamics may set the receptor activation threshold or drive active competition for co‐receptor binding. Fine‐tuning of these determinants of specificity fuels engineering efforts to broaden microbial recognition while maintaining sharp defence functionality (Tsitsikli et al., 2025). Structural biology has laid a solid groundwork for such efforts and has greatly advanced our understanding of how symbiotic and immunogenic signals are perceived in root endosymbiosis. Despite this progress, many LysM receptors remain insufficiently characterised (Figure 3b).

SymRK relays LysM symbiosis signal transduction, but whether RNS versus AM signalling specialisation is encoded within SymRK remains an open question. The different requirements for SymRK in epidermal cells, trichoblasts in legumes for rhizobia uptake and atrichoblasts for AM fungi may underlie this molecular specificity, in line with the proposed differences in CO sensitivity among these cell types (Genre et al., 2013). Addressing these questions, which are focussed on a single cell state rather than broad patterns across root tissues, has been challenging. In particular, the roles of LysM family members at the level of individual responsive cells have long been inaccessible. Spatial and single‐cell/nuclei transcriptomics have become increasingly feasible (Ferreras‐Garrucho et al., 2025; Frank et al., 2023; Pereira et al., 2025), perhaps soon to be supported by single‐cell proteomics. These techniques are anticipated to add new dimensions to our understanding of symbiotic signalling, allowing it to be dissected more precisely (Boxes 1 and 2).

Box 1 **Bullet point summary**

1. Symbiotic interactions in root endosymbiosis require a sophisticated host perception system that involves chitinous ligand recognition by members of the LysM‐RK family and downstream activity by SymRK.
2. AM fungi can likely be recognised by the plant host by the excess production of short‐chain chitin oligomers in response to SL.
3. The LysM receptor family forms diverse clades in land plants, with different characteristics, where LYK‐I and LYR clade receptors are required for root endosymbiosis.
4. Mechanisms that promote symbiotic signalling include slow ligand dissociation kinetics, enhanced by LysM receptor surface properties, increased abundance of short‐chain chitin oligomers, receptor competition and the assembly of higher order complexes.
5. Signal transduction downstream of LysM receptor complexes in AM and RNS converges on SymRK, which is required to initiate nuclear calcium oscillations through a phosphorylation‐dependent program.

Box 2 **Open questions**

1. Are COs and LCOs produced anabolically by AM fungi, or are they breakdown products, and how can the increase of CO4/5 be linked to the SL effect?
2. Are there differences in how plants recognise NFs, fungal COs and LCOs at different stages of infection, or in different tissues?
3. What are the precise spatiotemporal dynamics of secretion of LCOs and COs by rhizobia and AM fungi at different stages of symbiosis and accommodation, and how is this regulated?
4. Are there any other mechanisms that determine the specificity of AM symbiosis signalling, including potential molecules unique to AM fungi that have yet to be identified?
5. How is symbiotic signalling relayed from the plasma membrane to the nucleus to trigger calcium oscillations, and what specific molecular function does SymRK serve within this pathway?

## AUTHOR CONTRIBUTIONS

Conceptualisation and figure preparation: TF‐Ø and KP. Manuscript writing: TF‐Ø, MS and KP.

## CONFLICT OF INTEREST

The authors declare no conflicts of interest.

## Supporting information


**Figure S1.** Phylogenetic tree of lysine motif receptor‐like kinases in land plants.
**Figure S2.** Phylogenetic relationships of LysM‐RLK kinase sequences.
**Figure S3.** Ligand binding characteristics of the LYK receptor clade.
**Figure S4.** Ligand binding characteristics of the LYR receptor clade.
**Table S1.** Gene identifiers and common names for Lysine motif receptor‐like kinases in *Lotus japonicus* and *Medicago truncatula*.

## Data Availability

Source data, LysM protein sequences and multiple sequence alignment are available upon request.
