# Supplementary material for: Molecular recognition in plant root endosymbiosis
Source: Plant J. 2026 Aug 5;127(3):e71067. doi: 10.1111/tpj.71067 (PMC13441442; doi:10.1111/tpj.71067)
Supplement: Supplementary file 1 [file TPJ-127-0-s001.docx]

**Supplementary Figure 1. Phylogenetic tree of Lysine motif receptor-like kinases in land plants.**

Eleven clearly defined LYK and LYR clades could be annotated. In addition, a group of LYRs from non-seed plants can be seen, as well as gymnosperm LYRs that do not group within the main angiosperm LYR clades. The tree was built using IQtree (Minh et al., 2020) on sequences from 47 species based on orthogroups containing *L. japonicus* LysM-RLKs using OrthoFinder (Emms and Kelly, 2019) and visualised in iTOL (Letunic and Bork, 2024)*.* Truncated genes from the identified orthogroups were retained if they included a LysM domain. The tree is rooted at midpoint. Clades are highlighted with different colors and named based on (Buendia et al., 2018)**.**

**Supplementary Figure 2**. **Phylogenetic relationships of LysM-RLK kinase sequences.** Midpoint-rooted maximum-likelihood tree of kinase sequences from orthogroups containing *L. japonicus* LysM-RLKs. LYK and LYR clades are highlighted. The outer ring indicates proteins where LysM- or WAK-related domains were detected. Note the placement of LYS20 and LYS21 orthologs in the WAK receptor group. Numbers indicate bootstrap support values. Domains were annotated and extracted using hmmscan, and the tree was built using IQtree (Minh et al., 2020) and visualised in iTOL (Letunic and Bork, 2024)*.*

**Supplementary Figure 3. Ligand binding characteristics of the LYK receptor clade.**

Summarised are binding studies in which the dissociation constant (Kd) was estimated. LYK receptors bind chitin oligomers (COs), fungal lipochitooligosaccharides (Myc-LCOs), Nod Factors (NFs), exopolysaccharide (EPS) and β-glucans. Binding characteristics are shown in the Table. *Lj* – *Lotus japonicus*, *Mt* - *Medicago truncatula*. Methods: Surface Plasmon Resonance (SPR), Microscale Thermophoresis (MST) and Biolayer Interferometry (BLI). *Binding to EPS derived from *Mesorhizobium loti, Sinorhizobium meliloti* and *Rhizobium leguminosarum* was tested*,* highlighting the receptors’ promiscuity. ** - *Serendipita indica* β-glucans. References: (Bozsoki et al., 2020)^1^, (Broghammer et al., 2012)^2^, (Murakami et al., 2018)^3^, (Bozsoki et al., 2017)^4^, (Wong et al., 2020)^5^, (Kelly et al., 2023)^6^.

**Supplementary Figure 4. Ligand binding characteristics of the LYR receptor clade.**

Summarised are binding studies in which the dissociation constant (Kd) was estimated. LYR receptors bind chitin oligomers (COs), fungal lipochitooligosaccharides (Myc-LCOs) and Nod Factors (NFs). Binding characteristics are shown in the Table. *Mpa* – *Marchantia paleacea*, *Lj* – *Lotus japonicus*, *Mt* - *Medicago truncatula, Os – Oryza sativa, Bd –* *Brachypodium distachyon, Ta - Triticum aestivum, Nb –* *Nicotiana benthamiana, Pp - Prunus persica*. Methods: Surface Plasmon Resonance (SPR), Microscale Thermophoresis (MST), Biolayer Interferometry (BLI) and Isothermal Titration Calorimetry (ITC). * References: (Tan et al., 2025)^7^, (Gysel et al., 2021)^8^, (He et al., 2019)^9^, (Cullimore et al., 2023)^10^, (Ding et al., 2025)^11^, (Fliegmann et al., 2013)^12^, (Wang et al., 2023)^13^, (Gysel et al., 2025, preprint)^14^.

| **Clade name** | **Common name in *L. japonicus*** | **Gene ID** | **Common name in *M. truncatula*** | **Gene ID** |
| --- | --- | --- | --- | --- |
| LYK-I | NFR1 | *LotjaGi1g1v0062600* | LYK3 | *Medtr5g086130* |
| LYK-I | LYS1/NFRe | *NCBI:* *BAI79267* | LYK6 | *Medtr5g086040* |
| LYK-I | LYS2 | *LotjaGi1g1v0062700* | LYK1 | *Medtr5g086080 Medtr5g086090 Medtr5g086110 Medtr5g086120*  *Medtr5g086540*  *Medtr5g086660* |
| LYK-I | LYS6/CERK6 | *LotjaGi6g1v0196700_LC.1* | CERK1 (LYK9) | *Medtr3g080050* |
| LYK-I | LYS7 | *LotjaGi6g1v0290400* | LYK8 | *Medtr2g024290* |
|  |  |  |  |  |
| LYK-II | LYS3/EPR3 | *LotjaGi2g1v0324000* | LYK10 | *Medtr5g033490* |
| LYK-II | LYS8/EPR3a | *LotjaGi4g1v0157000* |  |  |
|  |  |  |  |  |
| LYK-III | LYS4 | *LotjaGi3g1v0021700* | LYK11 | *Medtr8g014500* |
| LYK-III | LYS5 | *LotjaGi3g1v0371400* |  |  |
|  |  |  |  |  |
| LYR-IA | NFR5 | *LotjaGi2g1v0394950* | NFP | *Medtr5g019040* |
| LYR-IA | LYS11 | *LotjaGi4g1v0114300* | LYR1 | *Medtr8g078300* |
| LYR-IB | LYS17 | *LotjaGi6g1v0000700* | LYR8 | *Medtr5g042440* |
|  |  |  |  |  |
| LYR-IIA | LYS16 | *LotjaGi1g1v0622300* | LYR10 | *Medtr7g029650* |
| LYR-IIB | LYS15 | *LotjaGi3g1v0488900* |  |  |
|  |  |  |  |  |
| LYR-IIIA | LYS12 | *Lj2g3v1828320* *NCBI: BAI79276* | LYR3 | *Medtr5g019050* |
| LYR-IIIB | LYS18 | *LotjaGi1g1v0417200* | LYR2 | *Medtr1g021845* |
| LYR-IIIC | LYS13 / CHIP13 | *Lj2g3v2899910*  *NCBI: BAI79277* | LYR4 | *Medtr3g080170* |
| LYR-IIIC | LYS14 / CHIP14 | *LotjaGi1g1v0065900*  *Lj2g3v2899900* |  |  |
|  |  |  |  |  |
| LYR-IV | LYS20/21 | *LotjaGi1g1v0482500*  *NCBI: BAI79282* | LYR5/LYR6 | *Medtr7g079320*  *Medtr7g079350* |

**Supplementary Table 1. Gene identifiers and common names for Lysine motif receptor-like kinases in *Lotus japonicus* and *Medicago truncatula*.**
